# Role of Fe/Co Ratio in Dual Phase Ce_0.8_Gd_0.2_O_2−δ_–Fe_3−x_Co_x_O_4_ Composites for Oxygen Separation

**DOI:** 10.3390/membranes13050482

**Published:** 2023-04-29

**Authors:** Liudmila Fischer, Ke Ran, Christina Schmidt, Kerstin Neuhaus, Stefan Baumann, Patrick Behr, Joachim Mayer, Henny J. M. Bouwmeester, Arian Nijmeijer, Olivier Guillon, Wilhelm A. Meulenberg

**Affiliations:** 1 Institute of Energy and Climate Research IEK-1, Forschungszentrum Jülich GmbH, 52425 Jülich, Germany; 2 Faculty of Science and Technology, Inorganic Membranes, University of Twente, P.O. Box 217, 7500 AE Enschede, The Netherlands; 3 Central Facility for Electron Microscopy GFE, RWTH Aachen University, 52074 Aachen, Germany; 4 Ernst Ruska-Centre for Microscopy and Spectroscopy with Electrons ER-C, Forschungszentrum Jülich GmbH, 52425 Jülich, Germany; 5 Institute of Energy and Climate Research IEK-12, Forschungszentrum Jülich GmbH Helmholtz-Institute Münster, 48149 Münster, Germany; 6 Jülich Aachen Research Allianc JARA-Energy, 52425 Jülich, Germany

**Keywords:** mixed ionic-electronic conductors, dual phase oxygen transport membrane, ceramic materials, optimization, microstructure, spinel-type ferrite, oxygen permeation

## Abstract

Dual-phase membranes are increasingly attracting attention as a solution for developing stable oxygen permeation membranes. Ce_0.8_Gd_0.2_O_2−δ_–Fe_3−x_Co_x_O_4_ (CGO-F(3−x)CxO) composites are one group of promising candidates. This study aims to understand the effect of the Fe/Co-ratio, i.e., x = 0, 1, 2, and 3 in Fe_3−x_Co_x_O_4_, on microstructure evolution and performance of the composite. The samples were prepared using the solid-state reactive sintering method (SSRS) to induce phase interactions, which determines the final composite microstructure. The Fe/Co ratio in the spinel structure was found to be a crucial factor in determining phase evolution, microstructure, and permeation of the material. Microstructure analysis showed that all iron-free composites had a dual-phase structure after sintering. In contrast, iron-containing composites formed additional phases with a spinel or garnet structure which likely contributed to electronic conductivity. The presence of both cations resulted in better performance than that of pure iron or cobalt oxides. This demonstrated that both types of cations were necessary to form a composite structure, which then allowed sufficient percolation of robust electronic and ionic conducting pathways. The maximum oxygen flux is j_O2_ = 0.16 and 0.11 mL/cm^2^·s at 1000 °C and 850 °C, respectively, of the 85CGO-FC2O composite, which is comparable oxygen permeation flux reported previously.

## 1. Introduction

Oxygen separation using oxygen transport membranes (OTMs) is a good alternative to cryogenic air separation for producing pure oxygen in oxyfuel combustion. These ceramic membranes are more economically beneficial than other known gas separation methods, consume less power, and produce high quality oxygen in a single step process. Therefore, there is increasing interest in the development and application of OTMs for industrial processes, e.g., for oxyfuel combustion and syngas production [1,2]. Mixed ionic-electronic conducting materials (MIEC) can be used for oxygen separation from air or the oxygen separation process can include exposure to flue gases containing CO_2_, SO_x_, dust, and steam [3,4,5]. In general, oxygen transport in dual- and multiphase membranes is implemented by using both ionic and electronic conducting phases. The presence of both of these phases is key to ensuring good percolation and thus performance of the material. This means that the concentrations of individual phase should be above the percolating threshold, which is generally reported as 30 vol.%. For dual-phase material, the ionic conductivity is normally the rate-limiting property. Accordingly, the main target in this field is to enable the reduction of the e-conducting phase without negatively affecting the final percolation properties of the material. Moreover, these materials should be sufficiently stable at the operating conditions, e.g., in flue gases at high temperatures and with an oxygen partial pressure gradient [6]. Since the fluorite:spinel ratio has a significant influence on performance [7], the 85:15 combination is chosen based on the previous investigations of the Ce_0.8_Gd_0.2_O_2−δ_–FeCo_2_O_4_ (CGO-FC2O) multi-phase material [8,9]. Additionally, as a reference, the 60:40 ratio is used, which is very common in the literature due to the high amount of the electron-conducting phase avoiding any problems with percolation.

To produce a reliable, mixed-conducting composite, the quality of the connectivity and compatibility of the two phases is crucial. Under real conditions, interconnectivity between particles is imperfect and can be dependent on many different factors. In addition, the surface can also contribute to the oxygen exchange process in an important, though more limited way [10,11]. Modifying the surface by applying a catalytic layer is required to maximize surface area and to eliminate all possible surface limitations.

Composites of acceptor-doped ceria in combination with electronic conducting materials are of great interest and have recently been widely investigated in the intermediate temperature range (600–850 °C) [2,4,12,13,14,15,16]. For the electron conducting material, oxides from aliovalent transition metals in particular iron and cobalt are well-studied. This includes perovskites revealing dependence on the microstructure, phase constituents, conducting properties as well as permeation performance [17,18,19]. Furthermore, the spinel system Fe_3−x_Co_x_O_4_ with x = 0, 1, 2, and 3 is of particular interest [20]. The mixed Fe-Co spinel structure is reported to have higher thermal stability compared to the normal Co_3_O_4_ spinel, illustrating that Fe substitution affects positively the thermal stability and stability at reducing atmospheres, which is advantageous for the potential application in membrane reaction. The functional properties of the pure Fe_3−x_Co_x_O_4_ system have been well-reported [20,21,22,23,24,25,26,27] and several members of that system were mixed with doped ceria forming dual- or multi-phase composites.

The solubility of the end member Co_3_O_4_ (CO) in CGO is less than 1%, and higher concentrations lead to Co_3_O_4_ segregation. With addition of >5 mol% of Co_3_O_4_ to the ceria, Co_3_O_4_ remains as a separate phase after sample preparation and sintering processes, and leads to an improved electrical conductivity. It has also been reported that a three-dimensional interconnection of the phases in a dual-composite forms only when the amount of cobalt oxide exceeds 10 mol%. Adding Co_3_O_4_ also increases the surface exchange coefficient effectively, increasing the connected transport pathways [27,28].

The addition of the other end member Fe_2_O_3_ (FO) often is used as a sintering aid for sample densification and, in some cases, can even reduce the sintering temperature by 100–150 °C [22,29,30]. When added to the CGO, iron oxide also has a positive effect on the total conductivity of CGO [22]. The presence of the iron in the CGO matrix reduces the lattice parameter of CGO [31]. The solubility of Fe_2_O_3_ in ceria reported in the literature ranges from 1 to 2%, which may be due to variations in preparation methods and operating temperatures [22,30,32]. It was also reported that the addition of FO to CGO increases the grain boundary and total conductivity of the material. High conductivity is expected due to the possible formation of a Schottky type potential barrier at the grain boundaries within the high temperature range. This can enhance electron transport along the grain boundaries but at the same time be an obstacle for ionic transport across them [33]. It also increases the density of materials and leads to grain growth [29,34].

The addition of FeCo_2_O [9,35] and Fe_2_CoO [15,36] has also been published revealing the significant phase interactions at sintering temperature. However, a systematic study of the role of Fe/Co-ratio on phase evolution and permeation performance is missing, because different fractions of ionic and electronic conducting phases in the composites were investigated.

Since the phase evolution in the composite CGO-FC2O (x = 2 in Fe_3−x_Co_x_O_4_) has been previously investigated in detail [33], three more members from the system Fe_3−x_Co_x_O_4_ with x = 0, 1, and 3, will be discussed here, i.e., Fe_2_O_3,_ Fe_2_CoO_4_ (F2CO), and Co_3_O_4_. Based on the previous work, nominal ceria/spinel ratios of 60:40 wt% and 85:15 wt% were selected, respectively, for microstructural and performance analyses. In the former case (60:40) good percolation of the electronic pathways is given, while the latter (85:15) is close to the percolation threshold.

## 2. Materials and Methods

### 2.1. Sample Preparation

Ce_0.8_Gd_0.2_O_2−δ_ (CGO) (Cerpotech, >99%,Tiller, Norway), Fe_2_O_3_ (FO) (Merck, 99%), and Co_3_O_4_ (CO) (Merck, 99%) powders used for the experiments were synthesized by the solid-state reactive sintering method (SSRS). Fe_2_O_3_ and Co_3_O_4_ were mixed, Fe:Co in a 2:1 ratio, resulting in spinel Fe_2_CoO_4_ (x = 1), which was added to commercially available ceria. Respective amounts of powders were weighed to create CGO-F2CO compositions with wt.% -ratios of 60:40, 80:20, and 85:15. Cobalt-free and iron-free composites were performed with ratios of 60:40 and 85:15. Over 48 h the powder mixtures were ball milled in polyethylene bottles using zirconia balls on a roller bench at a speed of 175 rpm. After drying for 48 h in ambient air at 70° C, powder mixtures were pressed using a uniaxial press in disc-shaped membranes with d = 20 mm and then sintered at 1200 °C for 5 h in air with a heating rate of 5 K min^−1^. At this sintering temperature the spinel partially reduces into a high temperature monoxide phase with a rock salt structure. To ensure complete reoxidation of the high temperature Co/Fe-monoxide phase to a spinel phase that is stable at operating temperatures (600–1000 °C), a slow rate of 0.5 K min^−1^ between 900 and 800 °C was implemented in the cooling cycle. This rate was determined by referring to the Fe_3−x_Co_x_O_4_ phase diagram. After the sintering step, all samples were ground down to 1 mm thick discs in 2 steps, applying SiC merge papers with different grits from P 800 to P 1200 (by WS FLEX 18C). On both sides of the discs, a porous La_0.58_Sr_0.4_Co_0.2_Fe_0.8_O_3−δ_ (LSCF) catalytic activation layer with a thickness of 5 µm was applied using a screen-printing technique. Discs were then calcined in the oven at 1100 °C for 5 h.

### 2.2. Characterization Methods

#### 2.2.1. Crystal Structure

Crystal structure was determined using the X-ray diffraction (XRD) diffractometer D4 ENDEAVOR (Bruker, Karlsruhe, Germany). Diffraction angle was chosen in the range of 2θ from 10° to 80°, with increments of 0.02° for 2θ and 0.75 s of measurement time per step. Analysis of the measured data was performed with X’Pert HighScore (PANalytical B.V., version 3.0.5) software. Phase quantifications and associated crystal structure analyses were carried out by Rietveld refinement using (Version 4.2.2) software. The errors of each fit were calculated individually and are reported in Table 1, Table 2 and Table 3.

#### 2.2.2. Microscopy

Scanning electron microscopy (SEM) and energy dispersive X-ray spectroscopy (EDXS) were used to obtain material morphology. SEM images were taken with a Zeiss Ultra 55 and a Zeiss Supra 50 VP1 (Carl Zeiss NTS GmbH, Oberkochen, Germany) SEM at different magnifications. The electronic conductivity of samples was enhanced by sputter deposition of a thin platinum layer prior to measurement.

For the transmission electron microscopy (TEM) analysis, all specimens were cut from composite pellets by focused ion beam (FIB) milling using an FEI Strata 400 system with a gallium ion beam. Further thinning and cleaning were performed using an Argon ion beam in a Fischione Nanomill 1040 at beam energies of 900 eV and 500 eV. TEM and energy-filtered TEM (EFTEM) imaging were performed using an FEI Tecnai F20 at 200 kV. For high-resolution high-angle annular dark-field (HAADF) imaging and EDXS chemical mapping, an FEI Titan G2 80–200 ChemiSTEM microscope equipped with an XFEG and a probe Cs corrector was used [37].

#### 2.2.3. Electrical Conductivity

The total conductivity of the single-phase perovskite samples was determined by analyzing the temperature-dependent impedance spectra with the help of a Novotherm HT 1200 frequency analyzer. All samples were coated with a Pt resinate paste (RP 070107, Heraeus GmbH, Hanau, Germany) and on both sides of the sample Pt sheet contacts were attached and measured in air. For all measurements, an AC voltage peak-to-peak amplitude of 40 mV was applied. As the electronic conductivity of both spinels was very high, no division into separate contributions from the grain bulk and grain boundary was visible. Nyquist plots instead only showed a straight line (ohmic contribution from e-conducting phase).

The temperature-dependent electrical (total) conductivity of the composites, which was also dominated by the electron conductivity of the e-conducting phase, was measured using a DC measurement setup (Keithley 2600B): the top of the sample pellet was in contact with a Pt microcontact with a diameter of about 400 nm and the bottom was in contact with a Pt sheet. Additionally, Pt resinate paste (RP 070107, Heraeus GmbH) was applied to the sheet to reduce contact resistance. By using light microscopy to measure the imprint on the sample, the exact size of the micro-contact was determined. This was then compared to the light microscopy images of the contact itself.

#### 2.2.4. Oxygen Permeation Measurements

The oxygen permeation tests of all materials were performed in a vertical quartz glass housing, where the composite membranes were sealed with two gold rings with inner diameters of 13 mm. The separation of the oxygen from ambient air fed with 250 mLN min^−1^ was performed between 650 °C and 1000 °C. Argon was used as a sweep gas with a 50 mLN min^−1^ flow rate using mass flow controllers (Bronkhorst, Kamen, Germany). A mass spectrometer (Omnistar, Pfeiffer Vacuum GmbH, Aßlar, Germany) detected the oxygen and nitrogen concentrations, in the permeate gas, i.e., oxygen enriched argon. Using the measured nitrogen concentration, the air leakage through either the membrane or the sealing was determined according to:

(1) $$j_{O_{2}}=F_{\mathrm{Ar}}\left(\frac{X_{O_{2}}-\frac{1}{4}X_{N_{2}}}{1-X_{O_{2}}-X_{N_{2}}}\right)\frac{1}{A_{\mathrm{mem}}}$$

with F_Ar_ being the argon flow rate (50 mLN min^−1^), $X_{O_{2}}$ and $X_{N_{2}}$ the oxygen and nitrogen concentration in the permeate gas, respectively, and Amem the open membrane area (1.33 mm^2^). The factor 1/4 reflects the O_2_/N_2_ ratio in the air feed, assuming that the leak is not gas selective.

The driving force of the permeation rate was not constant during the measurement, since the oxygen partial pressure in the permeate gas is temperature dependent. Additionally, the thickness of the discs after grinding deviates ±8% from the target thickness of 1 mm. Consequently, the driving force-normalized permeation rate, also referred to as permeance, was normalized to the reference thickness L_0_ = 1 mm and was calculated assuming Wagner behavior using the following equation

(2) $$\mathrm{Permeance}=\frac{j_{O_{2}}}{\ln\frac{p_{O_{2}}^{\prime }}{p_{O_{2}}^{\prime \prime }}}\frac{L_{\mathrm{mem}}}{L_{0}}$$

Here, $p_{O_{2}}^{\prime }$ and $p_{O_{2}}^{\prime \prime }$ are the oxygen partial pressures in the retentate and permeate gas, respectively, and L_mem_ is the actual membrane thickness. While the overall experimental error cannot be calculated precisely, it is assumed to be ±10%, which is well accepted in the literature.

## 3. Results and Discussion

### 3.1. Microstructure Evolution

The spinel system Fe_3−x_Co_x_O_4_ with the *Fd3m* structure was analyzed according to the Fe_3−x_Co_x_O_4_ phase diagram illustrated in Fe-Co phase diagramm from [38]. Generally, two spinel types are considered, iron-rich and cobalt-rich. The difference between the two spinel structures is the distribution of divalent and trivalent cations. To preserve the *Fd3m* symmetry in the normal spinel structure, divalent cations (Co^2+^) occupy all tetrahedral A sites, while all octahedral B sites are occupied by trivalent cations (Fe^3+^). This is in contrast to the inverse spinel structure, where the A sites as well as 50% of the B sites are occupied by trivalent cations, the remaining 50% of the B sites contain divalent cations [39,40,41]. Thus, the normal and inverse spinel structures can be expressed as AB_2_O_4_ and B(AB)O_4_, respectively [42]. There are three spinel regions on the Fe-Co phase diagram. Where concentrations of iron-doping are low, and a Co_3_O_4_-like structure is found; it has a normal spinel structure. At high iron-doping concentrations (x ≥ 1.2), conditions are favorable for the Fe_3_O_4_-like structure, which has an inverse spinel. In this case, only one spinel phase (inverse spinel) can be observed in the composite whose lattice parameter increases with the amount of incorporated iron. Pure Fe_3_O_4_ exists only at 1300 °C in accordance with the Fe-Co phase diagram. In the intermediate range (0.65 ≤ x ≤1.07), coexistence of both spinel types is possible with clear predominance of the inverse spinel structure [20]. This section may be divided by subheadings. It should provide a concise and precise description of the experimental results, their interpretation, as well as the experimental conclusions that can be drawn.

#### 3.1.1. Fe-Free Composites (x = 3 in Fe_3−x_Co_x_O_4_)

**Figure 1:**
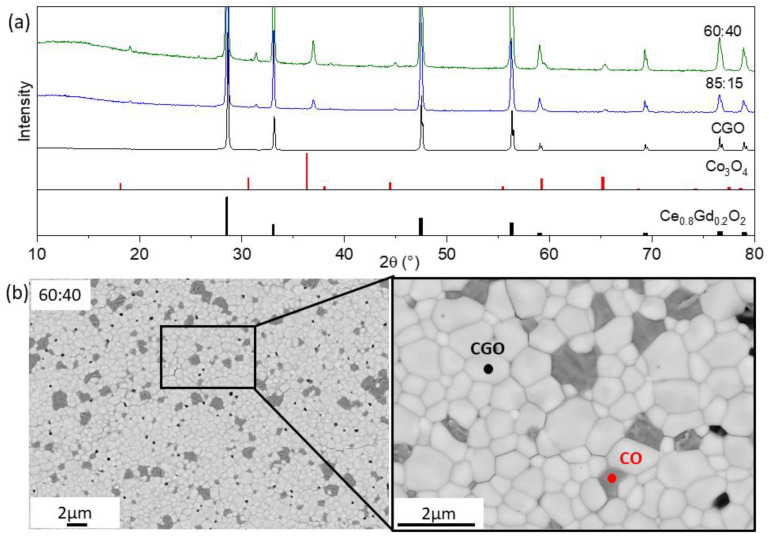
(**a**) The XRD patterns of CGO-CO materials with 60:40 (green) and 85:15 (blue) ratios sintered at 1200 °C for 5 h and single-phase CGO (black) sintered at 1400 °C for 5 h as well as peak positions of phases, (**b**) SEM image of CGO-CO morphology of the samples sintered at 1200 °C for 5 h.

Two iron-free composites with 60:40 and 85:15 ratios were subjected to microstructural and performance analyses (abbreviated later as 60CGO-CO and 85CGO-CO). During the fabrication and sintering steps, several problems appeared. The first sintering attempt under standard conditions of 1200 °C for 5 h resulted in all 60CGO-CO samples breaking and/or showing visual cracks as shown in Figure 1. This may have been due to the formation of an additional rock salt phase at 1200 °C, which is a known phenomenon in dual-phase materials [43]. In such a phase transformation, the volume expansion may cause the sample to crack. Macroscopic fractures were not observed for 85CGO-CO samples, but all samples had insufficient gas tightness.

Modifying the sintering programme to reduce the sintering temperature to 1100 °C with a 0.5 K/min cooling rate helped to reduce cracking, as according to the phase diagram, it helps the transformation of CoO_x_ to Co_3_O. However, it was still not possible to produce samples with sufficient gas-tightness for the permeation tests.

SEM analysis shows a mixture of the two phases in both composites, ceria (white) and cobalt oxide (dark grey). A mean grain size of 0.68 µm can be estimated for both phases from the SEM. In the 60:40 composite, intergranular cracks were detected along the grain boundaries, primarily between CGO and CGO grains, as can be seen in Figure 1b. The reason for the cracks is likely to be the internal stress resulting from either thermal expansion or the difference in chemical expansion of some localized CGO grains during sintering [43]. The character of the cracks in the 85:15 composite was slightly different. While fewer cracks were observed, they were found both along the grain boundaries as well as within the CGO grains.

Due to the low solubility of cobalt oxide (<1%), Co_3_O_4−δ_ was found as a separate phase in the samples [27,28]. It was also thermodynamically unfavorable to get gadolinium ions from the fluorite structure (CGO) and move them into the ABO_3_ phase structure at sintering temperature. Thus, perovskite Gd^3+^Co^3+^O_3_ phase formation is likely not possible at 1200 °C sintering temperature and instead only cobalt oxide is produced. The amount of added CO (15 and 40 wt.%) did not have much effect on grain size and phase distribution.

**Figure 2:**
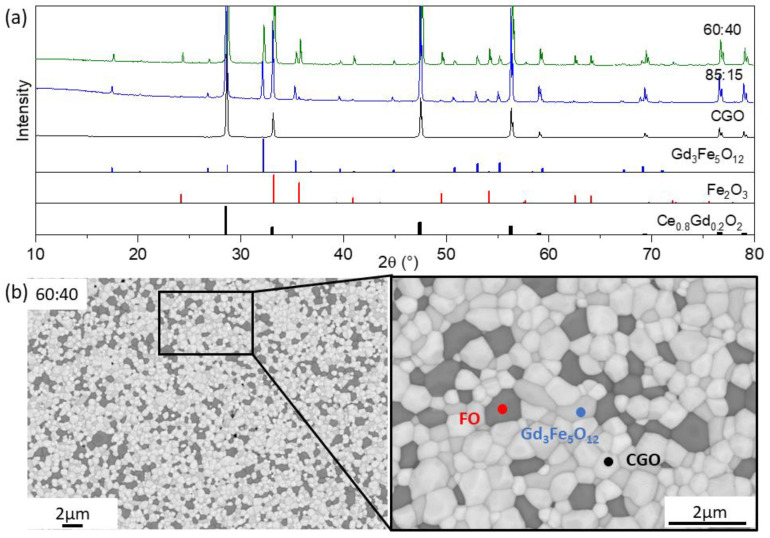
(**a**) The XRD patterns of the cobalt-free CGO-FO materials with 60:40 (green) and 85:15 (blue) ratios sintered at 1200 °C for 5 h and single-phase CGO (black) sintered at 1400 °C for 5 h as well as peak positions of phases. (**b**) SEM image of CGO-FO morphology of the sample sintered at 1200 °C for 5 h.

The presence of two phases was also confirmed by XRD analysis: CGO with cubic fluorite-type structure and Co_3_O_4_ with cubic spinel structure as shown in Figure 2a. With further Rietveld refinement, the main phase characteristics were analyzed. Weight percentages and lattice parameters for this series of composites are listed in Table 1. The CGO lattice parameter remained almost unchanged with the addition of CO to the acceptor-doped ceria, illustrating no phase interaction.

Other sintering attempts of the CGO-CO samples failed. Since most of the 60CGO-CO samples were broken at the fabrication stage, and most permeation tests were not successful, the composite was not used in further analyses.

#### 3.1.2. Co-Free Composite (x = 0 in Fe_3−x_Co_x_O_4_)

Similarly, two cobalt-free composites with 60:40 and 85:15 ratios were fabricated and subjected to microstructure analyses. The room-temperature XRD patterns revealed the presence of three main phases in both cobalt-free composites CGO-FO: CGO with a cubic fluorite structure, Fe_2_O_3_ with a rhombohedral structure, and cubic Gd_3_Fe_5_O_12_ (GCFO), as shown in Figure 2a.

Subsequent Rietveld refinement shows that, among these composites, the third phase accounted for 15–20 wt.%, listed in Table 2. The lattice parameter of CGO was reduced by 0.17% after addition of Fe_2_O_3_ to the acceptor-doped ceria, probably due to the substitution of smaller Fe^3+^ ions for Ce^4+^ in the fluorite matrix.

The XRD results were confirmed by SEM analysis, where three phases with a mean grain size of 0.5 µm were observed, as shown in Figure 2b: FO (dark grey), GCFO (light grey), and CGO (white). There were no visual defects or cracks after the sintering process.

In contrast to CO, the addition of FO to the nominal composite leads to a phase interaction during sintering at 1200 °C and the formation of a third phase Gd_3_Fe_5_O_12_. This is called gadolinium iron garnet, has a cubic structure, and is in the space group *Ia3d*, with respect to the Fe-Co phase diagram [44]. According to the literature, the solubility of iron oxides is a bit higher than Co_3_O_4_, which likely provides better compatibility with the phases in the CGO-FO composite. As a result, a Gd_3_Fe_5_O_12_-based phase is formed consisting of gadolinium cations from the fluorite phase as well as iron cations from the Fe_2_O_3_ phase. It is also likely that this phase contains traces of cerium cations on the A site. The third phase remains in the range of 15–20 wt.% and seems to be greatly affected by the amount of the FO in the nominal composite: the more FO the less Gd_3_Fe_5_O_12_ phase is formed. This tertiary phase was also observed and mentioned by Lin et al., who claimed that it had low conductivity compared to the perovskite-like structure [15]. According to these observations it can be concluded that the presence of iron cations in the composite is a necessary condition to form a tertiary phase during the sintering process, namely a cerium-doped Gd_3_Fe_5_O_12_ phase in a CGO-FO composite.

When comparing the fraction of the e-conducting phases in these composites with the permeation threshold, some assumptions were made. Since the exact composition and conducting properties of Gd_3_Fe_5_O_12_ were not clear, we could only assume that the Gd_3_Fe_5_O_12_phase provided some e-conducting properties to support e-transport [44,45,46]. In this case it was clear that while the permeation threshold of 30 vol.% was not reached by the CGO-CO composite, the CGO-FO composite reached this threshold at 17 wt.% FO.

#### 3.1.3. Iron Rich Spinel (x = 1 in Fe_3−x_Co_x_O_4_)

After investigating the composites with CO and FO, it was interesting to see how the combination of these two oxides in different proportions affected the microstructure and transport properties of dual-phase composites. The first candidate mix of Fe_2_O_3_ and Co_3_O_4_, where Fe/Co was held in a 1:2 ratio, resulting in a spinel FeCo_2_O_4_ (x = 2) phase has been previously published [9]. Here Fe_2_O_3_ and Co_3_O_4_ with Fe/Co in a 2:1 ratio, resulting in a spinel Fe_2_CoO_4_ (x = 1) phase was added into the ceria-based composite. In this study, three composite dual-phase membranes in combinations 60:40, 80:20, and 85:15 (abbreviated later as for example 60CGO-F2CO, etc.) were prepared using the SSRS method.

**Figure 3:**
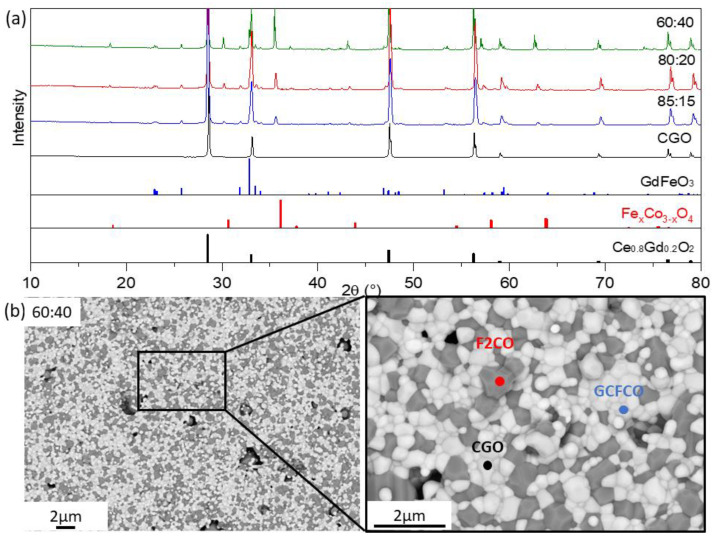
(**a**) XRD patterns of the three CGO-F2CO composites with 60:40 (green), 80:20 (red) and 85:15 (blue) ratios sintered at 1200 °C for 5 h and single-phase CGO (black) sintered at 1400 °C for 5 h as well as peak positions of fluorite, spinel, and perovskite phases; (**b**) SEM image of the surface dual-phase composite 60CGO-F2CO. White is CGO, light grey is GFO, and black is F2CO.

During the sintering process, several phase interactions took place which resulted in a mixture of the three phases. X-ray diffraction in Figure 3a clearly confirmed that all materials from the CGO-F2CO row had the following phases: CGO with a cubic fluorite structure, F2CO with a cubic spinel structure, and GdFeO_3_ with an orthorhombic perovskite structure. In the formation of the tertiary phase, approximately 10% of the gadolinium is taken from CGO and successfully incorporated at the A sites in the ABO_3_ structure [35]. For the B sites, iron is more favorably incorporated compared to cobalt. This perovskite phase may contain the cerium as well as cobalt traces.

Although all three phases can be distinguished by their X-ray diffraction patterns, the intensities of the spinel and perovskite phase peaks change with respect to the amount of nominal F2CO in the initial composite. In the SEM images in Figure 3b, where contrast of various phases can be distinguished, in combination with the further EDX analysis fluorite (white), spinel (dark grey), and perovskite phases (light grey) are found. These results are in a good agreement with the XRD.

Further Rietveld refinement quantified the composition of the dual-phase composites, which are listed in Table 3. In CGO-F2CO composite only one spinel type has been found. Similar to the CGO-FC2O (x = 2) composite described in [9], the weight fraction of the fluorite phase decreased as F2CO increased in the initial powder mixture.

The amount of GCFCO remains constant in the range of 12 ± 1 wt.%, regardless of the amount of F2CO in the nominal composite. Since the GdFeO_3_-based perovskite that is formed can be distinguished as a pure e-conductor according to [9], the increase in the total e-conducting phase in the composite occurs at the expense of the number of ion-conducting phases, which is the rate-limiting factor. The unit-cell parameters of the pure phases are known from the literature Fe_2_CoO_4_ (0.8385 nm) [47] and CGO (0.54209 nm) [48] and were measured before phase interaction. In our case, there was no remarkable change after SSRS and the sintering steps for the dual-phase material fabrication (F2CO: 0.837 nm, CGO: 0.5418 nm), as indicated Table 3. This indicated that we had an F2CO spinel structure in the composite after mixing the Fe_2_O_3_ and Co_3_O_4_ with Fe/Co in a 2:1 ratio.

**Figure 4:**
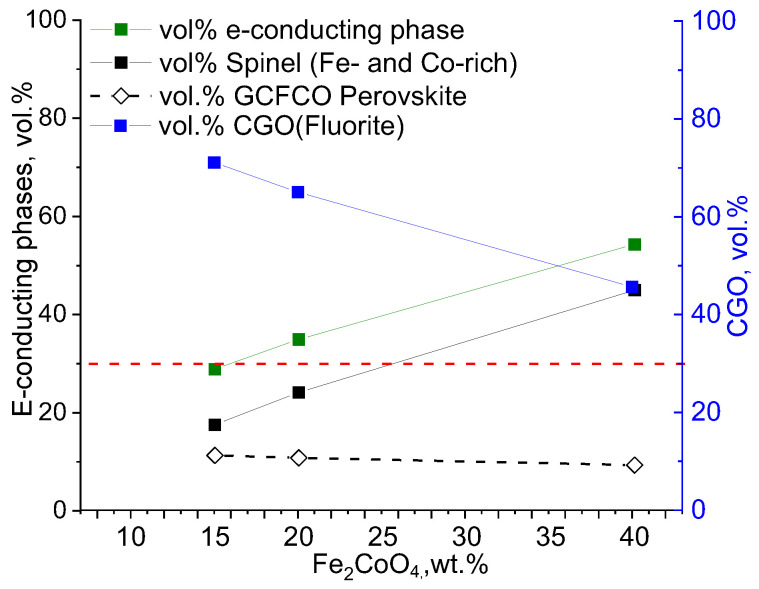
Volume fraction of detected phases in the CGO-F2CO composites.

Similar to the analysis performed on the CGO-FC2O (x = 2) composite, described in [9], the compositions of the three individual phases were also estimated. The resulting volume fractions of each phase are graphically represented in Figure 4.

**Figure 5:**
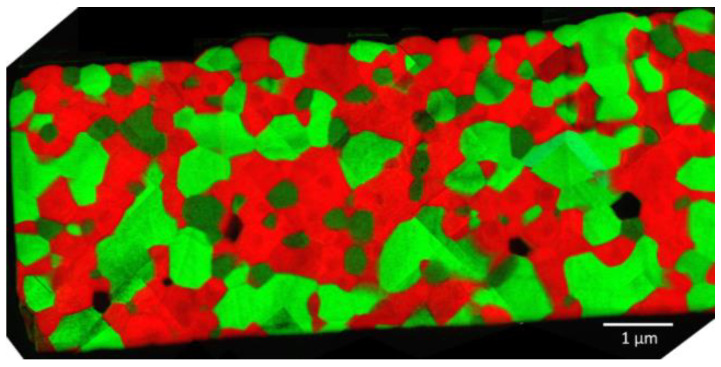
Distribution of the three phases: CGO (red), F2CO (light green) and GCFCO (dark green) in 60CGO-F2CO composite.

The percolation threshold of 30 vol.% (shown as a red dashed line in Figure 5) can be achieved at 25 wt.% of nominal F2CO in the composite (black line). Spinel and perovskite are responsible for e-transport, providing well-connected paths for the electrons in the material. The sum of the volume fraction of F2CO and GCFCO is shown in the graph in green. As can be seen that the threshold was reached already at 15 wt.% of the nominal F2CO in the composite material. This explains the relatively high oxygen flux for the CGO-F2CO (x = 1) composite material even at a low F2CO content (<20 wt.%).

**Figure 6:**
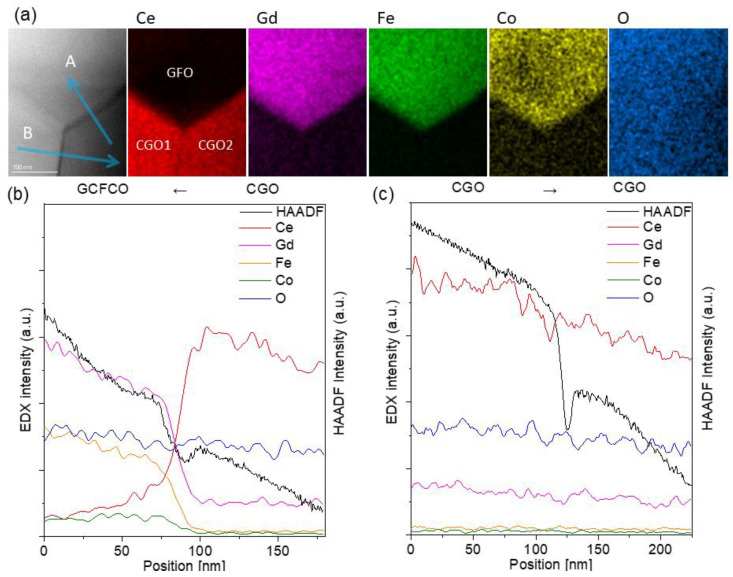
EDXS analysis of the grain boundaries. (**a**) The simultaneously acquired HAADF image and EDXS chemical mapping from Ce L, Gd L, Fe K, Co K and O K peak. (**b**) Line scans between CGO and GCFCO and (**c**) CGO and CGO grains.

Using TEM analysis, the presence of other phases was excluded. During the sintering process, the perovskite-like phase fraction was formed mostly between CGO grains, providing a well-connected network of the e-conducting phases and thus continuous pathways for electrons, which can be seen in Figure 6. The presence of this connected e-path helped to reach a sufficient percolation network in this composite and thus good oxygen flux.

The HAADF image and EDXS chemical mapping in Figure 6a were recorded around a junction of two CGO grains and one GCFCO grain, where the two phases could be clearly separated based on the elemental maps.

As indicated by the arrow labelled as A, intensity profiles across the CGO2 and GCFCO were extracted and then plotted in Figure 6b. All the elements smoothly transferred between the two phases, and no abrupt enrichment and/or depletion of any element was noticed.

Arrow B indicates the grain boundary between CGO1 and CGO2 grains, where also a smooth chemical transfer across the boundary can be observed.

In the CGO-F2CO composite, three different phases formed during the sintering process. This allowed the observation of six interface types, which resulted in different types of transport within the material. Interfaces between two spinel grains (F2CO-F2CO) and between spinel-orthorhombic (F2CO-GCFCO) as well as two orthorhombic (GCFCO-GCFCO) structures provided electronic transport, whereas CGO-CGO and CGO-GCFCO interfaces enhanced ionic transport.

**Figure 7:**
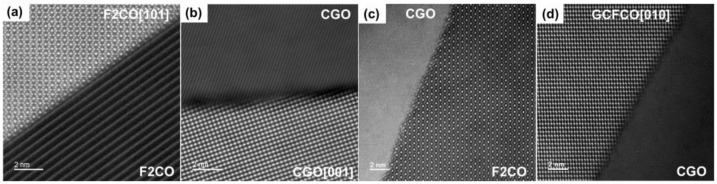
HAADF image of the grain boundary between different types of grains, (**a**) F2CO and F2CO, (**b**) CGO and CGO, (**c**) CGO and F2CO, and (**d**) CGO-GCFCO in the 60CGO-F2CO system.

Typical grain boundaries in the 60CGO-F2CO dual phase membranes are shown in Figure 7. All the grains are closely packed without any intergranular structures. Around the grain boundaries, the crystal structures of each grain are resolved down to the atomic scale, indicating no significant segregations or defects along the grain boundaries.

**Figure 8:**
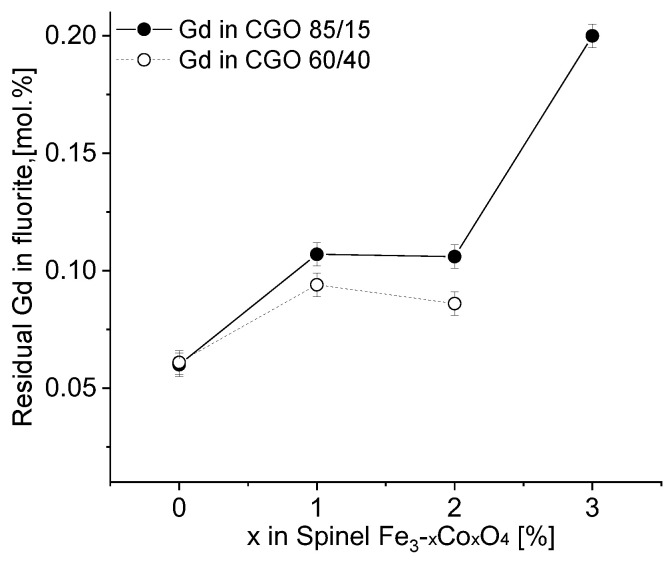
Residual gadolinium amount in fluorite dependent on Fe/Co ratio in CGO-based composites after sintering.

The influence of the Fe/Co ratio (x = 0–3) on the composite structure is investigated. The lattice parameter of pure CGO without phase interaction was 0.54246 nm and it was found to decrease after phase interaction due to Gd extraction (Table 1, Table 2 and Table 3). The residual gadolinium content in CGO (Figure 8) was calculated with help of the lattice parameter [9,49]. The iron-free composite (x = 3) reveals almost the same CGO lattice parameter compared to the pure CGO, because no phase interaction takes place. In contrast the pure Fe_2_O_3_ (x = 0) extracts most gadolinium so that only approx. 5 mol% remain in the ceria, which reduces its ionic conductivity [50]. A mixture of iron and cobalt (x = 1 or 2) a plateau with approx. 10 mol% residual gadolinium is observed, which shows still high ionic conductivity [50].

**Figure 9:**
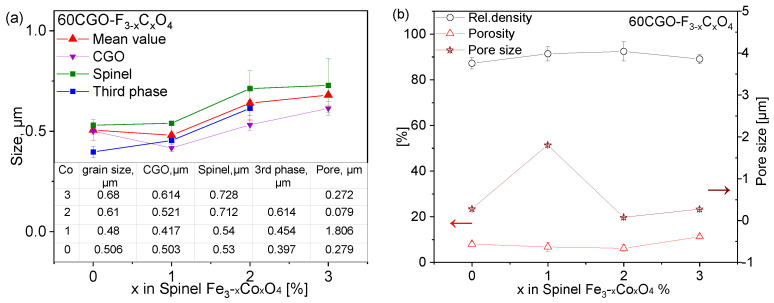
(**a**) The grain size and pore size dependency on the Fe/Co ratio in CGO20-based composite; (**b**) porosity and relative density dependency.

The grain size of each phase as well as the pore size is analyzed with ImageJ (1.8.0, USA) software on all samples [51] (Figure 9a). The spinel grains are slightly larger compared to the CGO and third phase, i.e., perovskite or garnet and all grain sizes slightly increase with increase in cobalt content x. The mean grain size value of the four composites is in the range of 0.48–0.68 µm.

The porosity (image analysis) does not vary significantly, which is in good agreement with the relative density values obtained geometrically (Figure 9b). The CGO-F2CO composite reveals a low number of larger pores, while CGO-FO has a higher number of pores with a small size << 1 µm. The highest densification > 91% relative densities was achieved with both F2CO and FC2O-added composites.

### 3.2. Permeation of the Composites

**Figure 10:**
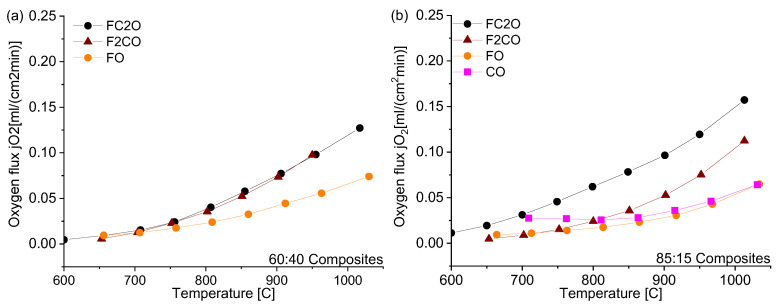
Oxygen permeation rates of the ceria-based materials for the (**a**) 60:40 and (**b**) 85:15 ratios.

Figure 10 shows the temperature-dependent oxygen permeation rates of the composites with nominal CGO-FCO wt-ratio of 60:40 and 85:15, respectively. In case of 60:40, the amount of the electronic conductor is high enough to ensure good percolation and the ionic conductivity of the ceria is rate-limiting. In consequence, the composites with the spinel phases, i.e., x = 1 and x = 2, show identical oxygen flux (Figure 10a). In contrast, the composite with pure iron oxide, x = 0, shows significantly lower flux because the resulting phases after sintering, i.e., Fe_2_O_3_ (hematite with corundum structure) and Gd_3_Fe_5_O_12_ (garnet structure) are known to have much lower electronic conductivity compared to the spinel and perovskite phases present in the other composites [15]. As described above, the composite with pure cobalt oxide, x = 3, could not be prepared and measured successfully.

**Figure 11:**
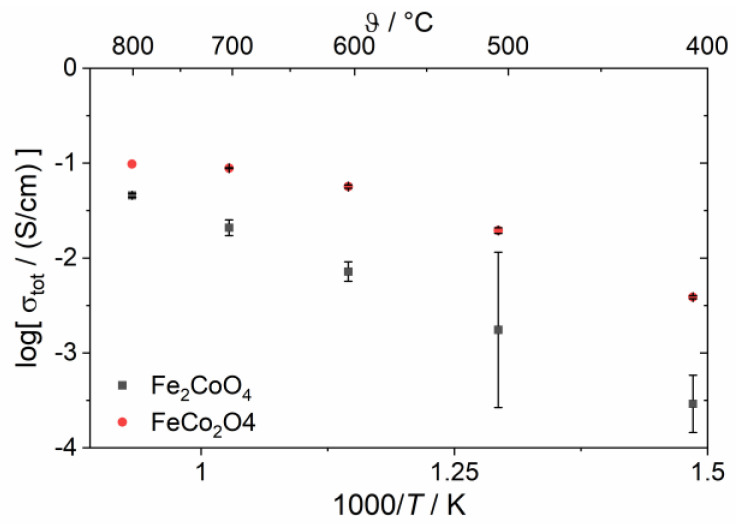
Total conductivity of spinel phases measured by impedance spectroscopy.

The amount of electronic-conducting phases in the composites with 85:15 nominal wt-ratio is around the percolation threshold as analyzed above. Now, the choice of the electronic conducting phase(s) becomes relevant and, thus, the oxygen fluxes vary significantly (Figure 10b). Again, the pure iron oxide (x = 0) shows the lowest performance due to lack of electronic conductivity. In case of Co_3_O_4_ (x = 3), the electronic conductivity is known to be high. However, since no phase interactions occur, the volume fraction of Co_3_O_4_ is too low to enable sufficient electronic transport. In consequence, the ambipolar conductivity and, thus, permeance is limited, too. The total (i.e., electronic) conductivity of FC2O measured by impedance spectroscopy is higher compared to F2CO as shown in Figure 11, which is in accordance with the literature [23]. Therefore, composites with FC2O (x = 2) show approx. 30% higher oxygen flux compared to that with F2CO (x = 1) at high temperatures. In addition, close to the percolation limit the phase distribution within the composite will have certain impact on its performance.

**Figure 12:**
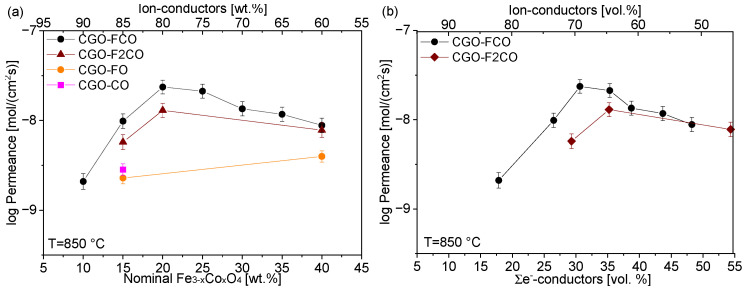
Permeance of the CGO-based composites depending on the Fe_3−x_Co_x_O_4_ content in (**a**) weight percent and (**b**) volume percent.

**Figure 13:**
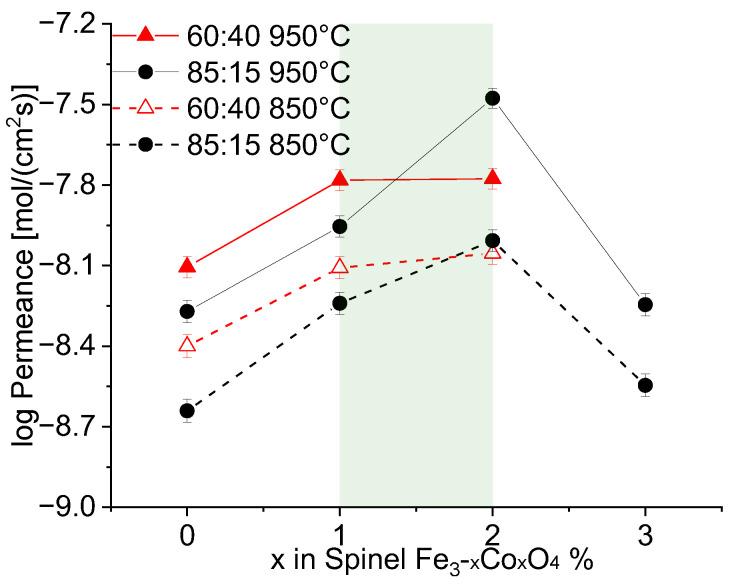
Dependency of the permeance on the cobalt amount in the composites with 60:40 and 85:15 ratios at T = 850 °C and 950 °C.

Permeance dependency on the e-conducting phase fracture of all studied composites is depicted in Figure 12. In summary, at high nominal spinel fraction in the composite, i.e., 40 wt%, the Fe/Co-ratio in the spinel phase plays a minor role for the permeance (Figure 13), because the ionic conductivity of the ceria phase is rate-determining. Hence, an improvement in the composites’ permeance can only be achieved by developing better ion conductors rather than electron conductors. However, pure iron oxide crystallizes in corundum structure rather than spinel structure providing significantly lower electronic conductivity and, thus, permeance. Addition of cobalt stabilizes the spinel structure, but pure cobalt oxide leads to cracking due to severe phase changes during sintering cycles.

At low nominal spinel fractions, i.e., 15 wt%, the Fe/Co-ratio in the spinel phase plays a significant role in determining its electronic conductivity. Still pure iron oxide shows poor performance. Pure cobalt oxide on the other hand is below the percolation threshold and, thus, does not provide sufficient electronic pathways. An enhanced permeance can be achieved in ceria-based composites by both improving electronic conductivity of the second phase as well as optimization of the microstructure, i.e., phase distribution, in order to provide sufficient electronic pathways. The best composite in this study was nominally composed of 85 wt% CGO and 15 wt% FC2O.

Direct comparison of published permeation data is in general difficult due to non-standardized sample geometry as well as measuring conditions. Nevertheless, the permeation rates of CGO-FCO composites investigated here are comparable to the literature data of other ceria-based membranes as shown in Table 4. This is expected because the permeation typically is limited by the ionic diffusion in the ceria phase in particular if percolation of both phases is guaranteed, e.g., commonly used 60:40 wt%. The actual electronic conductivity of the second phase is of minor importance as long as it remains significantly higher compared to the ionic one. Therefore, increasing the amount of ceria is a straightforward strategy for enhancing permeation rates provided that sufficient electronic conduction is maintained. Thus, close to the percolation limit the microstructure as well as the actual electronic conductivity of the second phase becomes increasingly important.

## 4. Conclusions

MIEC composites based on commercially available Ce_0.8_Gd_0.2_O_2−δ_ powder in combination with spinel Fe_3−x_Co_x_O_4_ (x = 0, 1, and 3) can be successfully synthesized using a cost-efficient solid state reactive sintering (SSRS) technique. During the sintering process, several phase interactions occur in CGO-FO (x = 0) and CGO-F2CO (x = 1) composites resulting in a mixture of three phases. For the cobalt-free composite, the CGO, FO, and the Gd_3_Fe_5_O_12_ garnet structure have been indicated, while in CGO-F2CO fluorite CGO, Fe-rich spinel, and a GdFeO_3_-based perovskite were detected. For x = 2 the spinel appears in two coexisting phases, which agrees with the phase diagram, while for x = 1 only one iron-rich spinel is indicated. In contrast, the composite CGO-CO (x = 3) has only two phases, CGO and Co_3_O_4_, and does not form a perovskite phase. In addition, it is difficult to produce crack-free samples of this composite. As a result, these iron-free and cobalt-free composites cannot be utilized as MIEC materials for use in an oxygen separation membrane.

A sufficient percolating network of ionic and electronic conducting phases in the investigated dual- (multi-)phase materials could only be achieved using both types of cations, iron and cobalt, fostering tertiary phase formation in the composite. This tertiary phase improved percolation and, accordingly, final permeance of material. Furthermore, the spinels Fe_2_CoO_4_ (x = 1) and FeCo_2_O_4_ (x = 2), made from Fe_3_O_4_ and Co_3_O_4_ in Fe/Co-proportions 2:1 and 1:2 respectively, provided adequate conductivity as a single phase. Consequently, the materials with FeCo_2_O_4_ (x = 2) and Fe_2_CoO_4_ (x = 1) remain the most attractive dual-phase materials for use as OTM. In the absence of one of the cations (iron or cobalt), a significant drop in oxygen permeance as well as limited stability is observed. The maximum oxygen flux is j_O2_ = 0.16 and 0.11 mL/cm^2^*s at 1000 °C and 850 °C, respectively, of the CGO-FC2O composite, which is comparable oxygen permeation flux reported previously. The main challenge in the development of dual-phase materials remains in maximizing the amount of the ionic conducting phase without losing percolation of the electronic conducting phase. Thus, the spinel system Fe_3−x_Co_x_O_4_ (1 < x < 2) presents a particularly high potential for high- and intermediate-temperature applications in the MIEC materials, ensuring adequate thermal stability and microstructural as well as transport properties. However, while the mechanical properties in air in particular for the CGO-FC2O composite are thoroughly studied [16,25,43], application-oriented testing, e.g., of chemical expansion, for targeted operation conditions is required.

## Figures and Tables

**Table 1:** Lattice parameter and fraction (F) of the phases after quantification by Rietveld refinement analyses of the CGO-CO composites sintered at 1200 °C for 5 h.

FCO	CGO		Co_3_O_4_	
wt.%	F, wt.%	a = b = c, Å	F, wt.%	a = b = c, Å
0	100.00	5.4246 [0]	0	-
15	83.50 [5]	5.4249 [0]	16.50 [5]	8.0808 [5]

**Table 2:** Lattice parameter and fraction (F) of the phases after quantification by Rietveld refinement analyses of the CGO-FO composites sintered at 1200 °C for 5 h.

FCO	CGO		Gd_3_Fe_5_O_12_		Fe_2_O_3_		
wt.%	F, wt.%	a = b = c, Å	F, wt.%	a = b = c, Å	F, wt.%	a = b, Å	c, Å
0	100.00	5.4246 [0]	0	-	0	-	-
15	74.30 [3]	5.4154 [5]	20.75 [2]	12.47 [2]	4.96 [2]	5.0351 [4]	13.7358 [0]
40	55.00 [3]	5.4155 [4]	15.33 [2]	12.47 [2]	30.7 [3]	5.0366 [6]	13.7359 [0]

**Table 3:** Lattice parameter and fraction (F) of the phases after quantification by Rietveld refinement analyses of the CGO-F2CO composites sintered at 1200 °C for 5 h.

FCO	CGO		F2CO		CGFCO			
wt.%	F, wt.%	a = b = c, Å	F, wt.%	a = b = c, Å	F, wt.%	a, Å	b, Å	c, Å
**0**	100.00	5.4246 [0]	0	-	0	-	-	-
**15**	74.00 [1]	5.4185 [1]	13.50 [1]	8.3688 [2]	12.30 [2]	5.3429 [8]	5.6099 [1]	7.658 [1]
**20**	69.00 [2]	5.4181 [8]	18.90 [1]	8.3699 [1]	12.00 [1]	5.3433 [8]	5.6097 [1]	7.662 [5]
**40**	51.60 [1]	5.4179 [4]	37.40 [9]	8.3835 [1]	11.00 [9]	5.3485 [2]	5.6103 [5]	7.669 [3]

**Table 4:** Comparison of the measured oxygen flux with the oxygen flux of the ceria-based dual-phase membranes reported in the literature.

Membrane Material	Weight Ratio	$j_{O_{2}}\;(\mathrm{mL}/\min/{\mathrm{cm}}^{2})$	T, (°C)	Thickness (mm)	Atmosphere	Synthesis	Coating	Ref.
Ce_0.8_Gd_0.2_O_2d_–FeCo_2_O_4_	85:15	0.16	850	1	Air/Ar	one-pot	+	[24]
Ce_0.8_Gd_0.2_O_2d_–CoFe_2_O_4_	60:40	0.1	850	1	Air/He	SSRS	+	[15]
Ce_0.9_Gd_0.1_O_2d_–Fe_2_O_3_	60:40	0.06	900	0.5	Air/He	Pechini		[52]
Ce_0.8_Gd_0.2_O_2d_–CoFe_2_O_4_	75:25	0.28	1000	1	Air/He	Pechini		[53]
Ce_0.8_Gd_0.2_O_2d_–FeCo_2_O_4_	85:15	0.11	850	1	Air/Ar	SSRS	+	[35]
Ce_0.8_Gd_0.2_O_2d_–Fe_2_CoO_4_	80:20	0.08	850	1	Air/Ar	SSRS	+	This work
Ce_0.8_Gd_0.2_O_2d_–Fe_2_CoO_4_	80:20	0.16	1000	1	Air/Ar	SSRS	+	This work
Ce_0.8_Gd_0.2_O_2d_–FeCo_2_O_4_	80:20	0.11	850	1	Air/Ar	SSRS	+	This work
Ce_0.8_Gd_0.2_O_2d_–FeCo_2_O_4_	80:20	0.20	1000	1	Air/Ar	SSRS	+	This work

## Data Availability

The data presented in this study are available on request from the corresponding author.
